# Volume, outcomes, and quality standards in thyroid surgery: an evidence-based analysis—European Society of Endocrine Surgeons (ESES) positional statement

**DOI:** 10.1007/s00423-020-01907-x

**Published:** 2020-06-10

**Authors:** Kerstin Lorenz, Marco Raffaeli, Marcin Barczyński, Leyre Lorente-Poch, Joan Sancho

**Affiliations:** 1 Department of Visceral, Vascular, and Endocrine Surgery, Martin-Luther University of Halle-Wittenberg, Ernst-Grube Strasse, 40 06120 Halle an der Saale, Germany; 2 U.O.C. Chirurgia Endocrina e Matabolica, Fondazione Policlinico Universitario Agostino Gemelli IRCCS, Rome, Italy; 3 Dipartimento Universitario di Medicina e Chirurgia Traslazionale, Università Cattolica del Sacro Cuore, Rome, Italy; 4 Department of Endocrine Surgery, Third Chair of General Surgery, Jagiellonian University Medical College, Kraków, Poland; 5 Secció del Servei de Cirurgia General de l’Hospital del Mar, Barcelona, Spain

**Keywords:** Thyroid surgery, Volume, Outcome, Quality, Recurrent nerve palsy, Hypoparathyroidism, Bleeding, Infection, Recurrence

## Abstract

**Introduction:**

Continuous efforts in surgical speciality aim to improve outcome. Therefore, correlation of volume and outcome, developing subspecialization, and identification of reliable parameters to identify and measure quality in surgery gain increasing attention in the surgical community as well as in public health care systems, and by health care providers. The need to investigate these correlations in the area of endocrine surgery was identified by ESES, and thyroid surgery was chosen for this analysis of the prevalent literature with regard to outcome and volume.

**Materials and methods:**

A literature search that is detailed below about correlation between volume and outcome in thyroid surgery was performed and assessed from an evidence-based perspective. Following presentation and live data discussion, a revised final positional statement was presented and consented by the ESES assembly.

**Results:**

There is a lack of prospective randomized controlled studies for all items representing quality parameters of thyroid surgery using uniform definitions. Therefore, evidence levels are low and recommendation grades are based mainly on expert and peer evaluation of the prevalent data.

**Conclusion:**

In thyroid surgery a volume and outcome relationship exists with respect to the prevalence of complications. Besides volume, cumulative experience is expected to improve outcomes. In accordance with global data, a case load of < 25 thyroidectomies per surgeon per year appears to identify a low-volume surgeon, while > 50 thyroidectomies per surgeon per year identify a high-volume surgeon. A center with a case load of > 100 thyroidectomies per year is considered high-volume. Thyroid cancer and autoimmune thyroid disease predict an increased risk of surgical morbidity and should be operated by high-volume surgeons. Oncological results of thyroid cancer surgery are significantly better when performed by high-volume surgeons.

## Introduction

Interpreting volume and outcome correlation in thyroid surgery is complex. First of all, looking at volume, the concept of “practice makes perfect” (numbers) rivals the concept of a selective referral to the (naturally) superior surgeon, thus generating higher volume. In addition, minimum numbers and centralization are influenced by diverse and conflicting players in the health system, namely insurances, government, and politics [1]. Data provided by the diverse players must always be suspected to serve a certain purpose, and oftentimes large data drawn from registries or health insurance companies is not risk stratified when providing outcome data, and the selected quality indicators may not be applicable or ideal. In the aim to improve outcome in thyroid surgery, the intent to reach perfection appears as applicable as minimizing the risk of avoidable harm. In general, the available literature published on volume and outcome aspects for thyroid surgery is compromised by low evidence and is dominated by USA-based data sources that may only partly be transferable to European health care systems. The dimensional range of surgeon volume provided in the current literature is considerate and the studies compare a wide range of definitions that in effect make comparison and meta-analysis error-prone. Also, when looking at hospital volume in thyroid surgery, it must be considered that this may but must not necessarily correlate strongly with surgeon volume. Keeping these premises in mind, it must be understood that to define minimal numbers in thyroid surgery remains scientifically low evidence-based and will not be accepted uncontested and may not apply to all types of health care systems.

For the present analysis, a literature search of PubMed was performed (31 December 2018) to retrieve articles published between 1 January 1990 and 31 December 2018. The following terms were used in the search text fields: thyroid surgery AND volume and outcome relationship OR benign thyroid surgery AND recurrent laryngeal nerve injury OR thyroid cancer surgery AND recurrent laryngeal nerve injury AND hypocalcemia OR hypoparathyroidism AND postoperative hemorrhage OR thyroid cancer surgery AND infection AND volume and outcome relationship AND thyroid cancer surgery.

Published observational and interventional studies that reported on volume and outcome relationship in thyroid surgery in humans were included. Reviews, letters, commentaries and editorials, articles with fewer than 100 patients, articles whose full text was not available in the English language, animal studies, irretrievable articles, and articles published before 1990 were excluded. Outcome measures included recurrent laryngeal nerve injury (transient and permanent), hypoparathyroidism (transient and permanent), bleeding, infection, completion thyroidectomy, local disease control, and recurrence rate.

The titles and/or abstracts of all retrieved articles were screened, and the full text of relevant articles reviewed for possible inclusion. Data collected included study and participants’ characteristics, type of surgery, and volume-outcome relationship. Missing data were excluded from the analysis. In the event of duplicate publication, only the publication with the most recent and complete data was included. Evidence levels (EL) and recommendation grades (RG) of the analyzed literature were assessed using adapted Sackett classification. In this, evidence levels (EL) descend from highest level of randomized controlled trials (ELI) and corresponding recommendation grades (RG), highest RGA, to lowest ELV and RGD for case reports or uncontrolled studies with little confidence in the estimated effect [2, 3].

The results of the analysis were presented and discussed at the 8th Conference of the European Society of Endocrine Surgeons (ESES), “Volumes, Outcomes, and Quality Standards in Endocrine Surgery” (Granada, Spain, May 16–18, 2019). This paper incorporates the results of the analysis of the literature and the outcomes of the discussions.

## Results

Results are overviewed in Table 4

### Recurrent laryngeal nerve injury in relation to surgeon volume

Risk of recurrent laryngeal nerve (RLN) injury in thyroid operations for various conditions depending on individual surgical volume (high-volume vs. low-volume surgeons) in different published series is shown in Table 1.

**Table 1:** Risk of RLN injury in thyroid operations for various conditions depending on individual surgical volume (high-volume vs. low-volume surgeons)

Author	Publication date	Patients	Definition operations/year		RLN injury	
			LVS	HVS	Odds ratio (95% CI)	*p*
Sosa [4] et al.	1998	5860 3954	< 30 < 10	> 100^a^ > 30^a^	0.69 (0.37–1.27) 0.42 (0.22–0.80)	0.235 0.009
Gourin [6] et al.	2010	21,270	< 30	> 30	0.46 (0.28–0.75)	0.002
Loyo [7] et al.	2013	871,644	< 10	> 23	0.71 (0.53–0.95)	0.024
Gonzalez-Sanchez [8] et al.	2013	225	< 40	> 40	0.07 (0.01–0.78)	0.029
Kandil [9] et al.	2013	10,081 21,625	< 10 < 10	> 100 > 30	0.53 (0.36–0.80) 0.73 (0.57–0.94)	0.002 0.017
Al-Quraysi [10] et al.	2016	77,863	< 30^b^	> 30	0.75 (0.62–0.90)	0.003
Adam [11] et al.	2017	16,954	< 25	> 25	0.68 (0.50–0.92)	0.010
Nouraei [13] et al.	2017	72,594	< 50	> 50	0.68 (0.52–0.88)	0.010

*LVS* low-volume surgeon; *HVS* high-volume surgeon

^a^In this study, surgical volume was calculated for the entire 6-year study period, not for caseload per year

^b^In this study, low-volume and intermediate-volume surgeons were defined as caseload 1–3 per year and 4–29 per year, respectively, but they were treated as altogether for the purpose of this calculation

In a statewide analysis of thyroidectomy performed in Maryland between 1991 and 1996, Sosa et al. reported that the individual surgeon’s experience rather than the hospital’s experience significantly correlated with complication rates. In this study that comprised 5860 patients, surgeons were categorized by volume of thyroidectomies over the 6-year study period: A (1 to 9 cases), B (10 to 29 cases), C (30 to 100 cases), and D (> 100 cases). Multivariate regression was used to assess the relation between surgeon caseload and in-hospital complications, length of stay, and total hospital charges, adjusting for case mix and hospital volume. After adjusting for case mix and hospital volume, highest-volume surgeons had the shortest length of stay (1.4 days vs. 1.7 days for groups B and C and 1.9 days for group A) and the lowest complication rate (5.1% vs. 6.1% for groups B and C and 8.6% for group A). Interestingly, prevalence of RLN injury was significantly reduced in operations performed by high-volume surgeons (groups C+D) who performed more than 30 operations per 6-year study period when compared with the lowest-volume surgeon (group A) who performed less than 10 operations per 6-year study period (OR 0.42; 95% CI 0.22–0.80; *p* = 0.009) [4] (ELIII, RGD).

Gourin et al. analyzed 21,270 thyroid surgical procedures performed by 1034 surgeons at 51 hospitals in Maryland between 1990 and 2009. High-volume surgeons (25 or more thyroid operations per year) were more likely to perform total thyroidectomy (OR 2.50; *p* < 0.001) and neck dissection (OR 1.86; *p* < 0.001), had a shorter length of hospitalization (OR 0.44; *p* < 0.001), and had a lower incidence of recurrent laryngeal nerve injury (OR 0.46; *p* = 0.002), hypocalcemia (OR 0.62; *p* < 0.001), and thyroid cancer surgery (OR 0.89; *p* = 0.01). In addition, compared with intermediate- and low-volume surgeons, high-volume surgeons were significantly more likely to perform total thyroidectomy procedures and neck dissection. High-volume surgeons were associated with increased case complexity scores, reflecting the presence of advanced comorbid disease, intensive care unit utilization, and decreased length of hospitalization [6] (ELIII, RGC).

Loyo et al. analyzed discharge data from the Nationwide Inpatient Sample for 871,644 patients who underwent surgery for thyroid disease in 1993 through 2008 using cross-tabulations and multivariate regression modeling. High-volume surgeons (> 23 operations per year) were significantly more likely to perform total thyroidectomy (odds ratio (OR) 1.4; *p* < 0.001) and had a lower incidence of RLN injury (OR 0.7; *p* = 0.024), hypocalcemia (OR 0.7; *p* = 0.002), and in-hospital death (OR 0.3; *p* = 0.004). High-volume hospital care (> 76 operations per year) was not associated with extent of surgery, postoperative morbidity, or mortality after adjusting for surgeon volume. After controlling for other variables, thyroid surgery in 2001 through 2008 was associated with an increase in cases performed by low-volume (relative risk ratio (RRR) 1.5; *p* < 0.001), intermediate-volume (RRR 1.7; *p* < 0.001), and high-volume surgeons (RRR 2.1; *p* < 0.001), high-volume hospitals (RRR 2.0; *p* = 0.008), total thyroidectomy (RRR 2.1; *p* < 0.001), and neck dissection (RRR 1.3; *p* = 0.016) [7] (ELIII, RGC).

Morbidity following thyroid surgery (permanent RLN palsy) was also less frequent among patients operated on by endocrine-dedicated surgeons vs. general surgeons in a prospective cohort study published by Gonzalez-Sanchez et al. (OR 0.07; 95% CI 0.01–0.78; *p* = 0.029) [8] (ELIII, RGD).

Kandil et al. used the nationwide inpatient sample to identify all patients who underwent total thyroidectomy between 2000 and 2009 and identified 46,261 procedures. In this study, the effects of surgeon volume and hospital characteristics on predicting patient outcomes were analyzed. A high-volume surgeon (> 100 operations per year) was in this study a predictor of diminished risk of RLN injury (OR 0.53; 95% CI 0.36–0.80; *p* = 0.002). In addition, the protective effect on prevalence of RLN injury was maintained in operations performed by intermediate-volume (30–100 operations per year) surgeons (OR 0.73; 95% CI 0.57–0.94; *p* = 0.017) [9] (ELIII, RGC).

Al-Qurayshi et al. performed cross-sectional analysis of adult inpatients in US community hospitals using the Nationwide Inpatient Sample for the years 2003 through 2009 that underwent thyroidectomy and identified 77,863 individuals who served as a study group. A high-volume surgeon was a predictor of diminished risk of RLN injury when compared with the low- and intermediate-volume surgeon (OR 0.75, 95% CI 0.6–0.90; *p* = 0.003) [10] (ELIII, RGC).

Exact threshold number of cases defining a “high-volume” thyroid surgeon remains inconsistent in published series. Adam et al. used a multivariate logistic regression model with restricted cubic splines to estimate the association between annual surgeon volume and incidence of the complications. This statistic model draws a flexible function with robust behavior at the tails of predictor distributions. The use of this model was aimed to fix the range of annual surgeon volumes corresponding to a change in the relative log odds of experiencing any postoperative complication.

This is the first study in which a statistical method has been used in order to establish activity volume threshold. Adam et al. addressed this issue in a study comprised 16,954 patients undergoing total thyroidectomy and including 47% patients with thyroid cancer and 53% with benign thyroid disease. Median annual surgeon volume was 7 cases and 51% of surgeons performed 1 case per year. After adjustment, the likelihood of experiencing a complication decreased with increasing surgeon volume up to 26 cases per year (*p* < 0.01). Among all patients, 81% had surgery by low-volume surgeons (≤ 25 cases/year). With adjustment, patients undergoing surgery by low-volume surgeons were more likely to experience complications (OR 1.51; *p* = 0.002) and longer hospital stays (+ 12%; *p* = 0.006). Patients had an 87% increase in the odds of having a complication if the surgeon performed 1 case per year, 68% for 2 to 5 cases per year, 42% for 6 to 10 cases per year, 22% for 11 to 15 cases per year, 10% for 16 to 20 cases per year, and 3% for 21 to 25 cases per year. A high-volume surgeon (> 25 operations per year) was in this study a predictor of diminished risk of RLN injury (OR 0.68; 95% CI 0.50–0.92; *p* = 0.01) [11] (ELIII, RGC).

Nouraei et al. analyzed recently national trends, outcomes, and volume-outcome relationships in thyroid surgery in a large cohort of 72,594 patients who underwent elective thyroidectomy in the UK between 2004 and 2012. Most patients in this study underwent hemithyroidectomy (51%) or total thyroidectomy (32%). Patients underwent surgery for benign (52.5%), benign inflammatory (21%), and malignant (17%) thyroid diseases. Increased surgeon volume significantly reduced lengths of stay: the proportion of length of stay outliers fell from 11.8% for patients of occasional thyroidectomists (< 5 per year) to 2.8% for patients of high-volume surgeons (> 50 thyroidectomies a year). High-volume surgeons had a reduced incidence of vocal cord palsy (OR 0.68; 95% CI 0.52–0.88; *p* = 0.01), and volumes > 30 were consistently protective. Hence, based on outcomes of this study, high-volume surgeon was defined as a surgeon who performs 50 or more thyroidectomies per year and achieves lower complications and shorter lengths of stay [13] (ELIII, RGD).

Similar conclusions were recently drawn by Melfa et al. who published a systematic review focused on surgeon volume and hospital volume in endocrine neck surgery with special emphasis put on how many procedures are needed for reaching a safety level and acceptable costs. Some differences in outcomes between these investigated categories were underlined: best results of the high-volume surgeon were evident especially in terms of complications, and on the contrary, best outcomes of a high-volume center were mainly economics, such as lower hospital stay and general costs of the procedures. A cut-off of 35–40 thyroidectomies per year for single surgeon appears reasonable for identifying an adequate high-volume practice [16] (ELIII, RGD).

### Recurrent laryngeal nerve injury in relation to hospital volume

Minimum hospital volume needed for reaching a satisfying level of safety is less clear and ranges from 20 to 200 thyroid operations per year in published series. However, a cut-off of 90–100 thyroidectomies for a single center appears reasonable for identifying an adequate high-volume practice [16] (ELIII, RGD).

Mitchell et al. retrospectively analyzed 395 reoperative thyroid and parathyroid surgeries at a tertiary care hospital from 1999 to 2007. In this study, public discharge data were used to classify hospitals as low-volume hospitals (below 20 cases per year) or high-volume hospitals (20 or more cases per year). Operations performed at low-volume hospitals resulted in RLN injury in 9% of patients which was significantly higher than seen after operations performed at high-volume hospitals, where 3% of patients suffered RLN injuries (*p* < 0.05) [15] (ELIV, RGD).

Lifante et al. retrospectively analyzed data of 20,140 patients who underwent thyroid surgery in hospitals in the Rhône-Alpes area of France between 1999 and 2004, including 4006 procedures for thyroid cancer. Compared with hospitals performing a high volume of procedures for all thyroid diseases (100 or more operations per year), the risk of a unilateral procedure for thyroid cancer increased by 2.46 (95% CI 1.63–3.71) in low-volume hospitals (less than 10 operations per year) and by 1.56 (95% CI 1.27–1.92) in medium-volume centers (10–99 operations per year) [17] (ELIV, RGD).

Thomusch et al. analyzed risk factors for postoperative complications of benign goiter surgery in a prospective multicenter study comprised 7266 patients operated on in 45 East German hospitals from January 1 through December 31, 1998. In this study, outcomes were stratified to the institutional annual volume: high-volume providers (> 150 operations per year) performed 69% (5042/7266), intermediate-volume (50–150 operations per year) providers 27%, and low-volume (< 50 operations per year) providers 4% (258/7266) of operations. In logistic regression analysis, hospitals with an operative volume of fewer than 50 operations per year had a 1.3-fold increased risk for transient RLN palsy (*p* < 0.05) in spite of a trend that small-volume providers tended to perform more operations for uninodular goiter and high-volume providers treated more patients with Graves’ disease and recurrent goiter [5] (ELIII, RGC).

Liang et al. retrospectively analyzed a cohort of 125,037 thyroidectomy patients treated at Taiwan hospitals from 1996 to 2010 with special focus paid on relationships between hospital/surgeon volume and patient outcomes using propensity score matching. In this study, both high-volume hospitals and high-volume surgeons were associated with significantly shorter length of stay and lower costs compared with their low-volume counterparts (*p* < 0.001). However, different volume groups had similar in-hospital mortality rates. Unfortunately, morbidity was out of the scope of this study [12] (ELIII, RGC).

### Recurrent laryngeal nerve injury in operations for thyroid cancer vs. benign thyroid disease

Risk of RLN injury in thyroid surgery for cancer vs. other benign conditions in different published series is shown in Table 2. Surgery for thyroid cancer was a predictor of increased risk of RLN injury in many published large cohort series and this increased risk was estimated to vary between 1.8-fold and 2.9-fold (*p* < 0.001) 5 (ELIII, RGC), 6 (ELIII, RGD), 11 (ELIII, RGD), 17 (ELIII, RGC).

**Table 2:** Risk of RLN injury in thyroid surgery for cancer vs. other benign conditions

Author	Publication date	Patients	RLN injury in thyroid cancer surgery	
			Odds ratio (95% CI)	*p*
Dralle [18] et al.	2004	16,448	2.04 (1.41–2.96)	< 0.001
Gourin [6] et al.	2010	21,270	2.90 (2.14–3.94)	< 0.001
Loyo [7] et al.	2013	871,644	1.99 (1.71–2.32)	< 0.001
Nouraei [13] et al.	2017	72,594	1.80 (1.51–2.14)	< 0.001

*RLN* recurrent laryngeal nerve

### RLN injury in operations for autoimmune thyroid disease vs. multinodular goiter

Uncertainty exists concerning the real incidence of thyroid surgery-specific complications after operations for autoimmune thyroid disease (AITD) [19] (ELIII, RGC). However, most authors maintain that AITD (including Graves’ disease and Hashimoto’s thyroiditis) carries a high risk for RLN palsy and hypoparathyroidism, resulting in complication rates of 4.0–12.3% and 3.8–14.0%, respectively [19] (ELIII, RGC).

Kandil et al. used the nationwide inpatient sample to identify all patients who underwent total thyroidectomy between 2000 and 2009 and identified 46,261 procedures. In this study, the effects of surgeon volume and hospital characteristics on predicting patient outcomes were analyzed. In addition, univariable and multivariable analyses were used to examine the effects of the indication for surgical care on postoperative outcomes. Patients with Graves’ disease had the highest postoperative complications (17.5%) compared with patients undergoing total thyroidectomy for other benign (13.9%) and malignant (13.2%) thyroid disease (*p* < 0.001). After stratification by surgeon volume, Graves’ disease was found to be a significant predictor of postoperative complications in surgeries performed by low-volume and intermediate-volume surgeons (OR 1.39; 95% CI 1.08–1.79; *p* = 0.01 and OR 1.34; 95% CI 1.06–1.69; *p* = 0.02, respectively). However, Graves’ disease was not a significant predictor of postoperative complications when performed by high-volume surgeons (OR 1.07; 95 %CI 0.62–1.83; *p* = 0.81). Hospital volume had an inconsistent and marginal protective effect on postoperative outcomes [9] (ELIII, RGC). Unfortunately, the issue of RLN injury was not stratified to surgical indications for surgery in this study. However, prevalence of postoperative vocal fold paralysis was significantly associated with surgical volume and both low-volume and intermediate-volume surgeons had higher risk of vocal fold paralysis than high-volume surgeons (1.5% vs. 1.2% vs. 0.8%, respectively; *p* = 0.009) [9] (ELIII, RGC). Hence, authors concluded that surgery for Graves’ disease is associated with a higher risk for complications when performed by less experienced surgeons. This finding should prompt recommendations for increasing surgical specialization and referrals to high-volume surgeons in the management of Graves’ disease.

Thomusch et al. published recently a prospective multicenter European study focused on prevalence thyroid surgery-specific complications among patients with AITD (*n* = 2488) vs. multinodular goiter (*n* = 16,467) and utilizing logistic regression analysis to evaluate risk factors for transient and permanent RLN palsy and hypoparathyroidism. The rate of temporary and permanent vocal cord palsy ranged from 2.7 to 6.7% (*p* = 0.623) and from 0.0 to 1.4% (*p* = 0.600) among institutions involved, respectively. In logistic regression analysis of transient and permanent vocal cord palsy, AITD was not an independent risk factor for RLN injury (data shown in Table 3). Hence, it is evidence-based that surgery for AITD is safe in comparison with surgery for multinodular goiter in terms of general complications and RLN palsy [19] (ELIII, RGC). However, it should be underlined that surgeons who contributed to this study were recruited among endocrine surgeons practicing in 68 hospitals in 6 European countries, and all of them were high-volume thyroid surgeons (100 or more thyroid operations per year). Hence, outcomes reached in this study cannot be universally extrapolated to results of thyroid surgery at hands of low-volume and intermediate-volume surgeons.

**Table 3:** Risk of RLN injury in autoimmune thyroid disease vs. benign multinodular goiter (data from PETS 2 study) [18]

RLN injury	MNG *n* = 16,467			Graves’ disease *n* = 1141			Hashimoto thyroiditis *n* = 1268		
	%	HR (95% CI)	*p*	%	HR (95% CI)	*p*	%	HR (95% CI)	*p*
Transient	3.9	1		3.4	0.86 (0.68–1.10)	0.238	3.5	0.69 (0.22–2.18)	0.691
Permanent	0.8	1		0.5	0.66 (0.35–1.21)	0.174	0.9	1.77 (0.35–8.94)	0.481

*MNG* multinodular goiter; *RLN* recurrent laryngeal nerve

### Propositional summary recurrent laryngeal nerve injury and volume

1. Surgeon volume and outcome relationship exists in thyroid surgery with respect to prevalence of RLN injury. A cut-off value of > 50 thyroidectomies for a single surgeon per year appears reasonable for identifying a high-volume surgeon.
2. Hospital volume and outcome relationship is less clear than surgeon volume in thyroid surgery with respect to prevalence of RLN injury. However, a cut-off value of 100 thyroidectomies for a single center per year appears reasonable for identifying an adequate high-volume unit.
3. Surgery for thyroid cancer is a predictor of increased risk of RLN injury in the pooled cohort of surgical volume.
4. Surgery for AITD is a predictor of increased risk of RLN injury in the pooled cohort of hospital volume.
5. Current evidence suggests that total thyroidectomy for thyroid cancer or AITD should be undertaken by high-volume surgeons who have lower RLN injury rates than low-volume surgeons.

### Hypocalcaemia/hypoparathyroidism in relation to surgeon volume and experience and hospital volume

In a statewide analysis of thyroidectomy performed in Maryland between 1991 and 1996, hypoparathyroidism rate was low, 0.3% in average [4] (ELIII, RGC). Incidence of post-thyroidectomy hypocalcaemia was not significantly different across volume groups [4]. The authors interpreted such unexpected finding as a consequence of the fact that calcium levels were not measured during hospitalization in all the patients, and most cases of hypocalcaemia were diagnosed after discharge and were not included in the analysis.

In a prospective multi-institutional study including 45 hospitals and 7266 patients operated for benign goiter, Thomusch et al. (ELIII, RGC) found that transient and permanent hypoparathyroidism occurred in 6.4% and 1.5% of the cases, respectively. There were no significant differences among hospital groups with different operative volumes [5] (ELIII, RGC).

In a statewide analysis of 21,270 thyroidectomies performed in Maryland between January 1, 1990 and July 1, 2009, postoperative hypocalcaemia was significantly less likely for patients operated by high-volume surgeons (> 100 thyroid procedures per year) (OR 0.49; 95% CI 0.41–0.57; *p* < 0.001) [4] (ELIII, RGC). Hospital volume (high volume > 150 thyroid procedures per year) had no impact on postoperative hypocalcaemia rate.

Using discharge data from the Nationwide Inpatient Sample for 871,644 patients who underwent thyroid surgery between 1993 and 2008, Loyo et al. (ELIII, RGC) found that postoperative hypocalcaemia was significantly less likely for patients operated by high-volume (> 23 thyroid procedures per year) and intermediate-volume (9–23 thyroid procedures/year) (OR 0.63; 95% CI 0.50–0.79; *p* < 0.001 and OR 0.86; 95% CI 0.76–0.97; *p* = 0.031, respectively), after controlling for other variables. Similarly, postoperative hypocalcaemia was significantly less likely in patients undergoing thyroidectomy at high-volume hospital (annual case load > 76) (OR 0.72; 95% CI 0.54–0.97; *p* = 0.031) [7]. However, after adjusting for surgeons’ volume, hospital volume had no impact on hypocalcaemia rate.

The fact that postoperative hypocalcaemia and hypoparathyroidism are related more to surgeon volume than to hospital volume was confirmed by a single-institution study comparing post-thyroidectomy complications between low-volume non-dedicated surgeons (< 5 procedures per year) and high-volume endocrine surgeons (> 40 procedures per year) [7] (ELIII, RGC). Permanent hypoparathyroidism (hypocalcaemia lasting > 1 year) was significantly less likely in patients operated by high-volume specialized surgeons (3/130 vs. 3/16, OR 8.12; 95% CI 1.51–43.7, *p* = 0.01).

In the study of Kandil et al. (ELIII, RGC) over 46,261 procedures, the effects of surgeon volume and hospital characteristics on predicting patient outcomes were analyzed. High-volume surgeons (> 100 operations for year) had a lower rate of post-thyroidectomy hypocalcaemia (4.7%) with respect to low-volume surgeons (< 30 procedures per year) (hypocalcaemia rate 12.1%) and to intermediate-volume surgeons (9.4%) (*p* < 0.01) [9].

Al-Qurayshi et al. (ELIII, RGC) performed cross-sectional analysis of adult inpatients who underwent thyroidectomy in US community hospitals using the Nationwide Inpatient Sample for the years 2003 through 2009 and identified 77,863 individuals who served as a study group. Thyroidectomy performed by a high-volume surgeon was a predictor of diminished risk of hypocalcaemia when compared with low- (OR 1.43, CI 0.95–2.17; *p* = 0.09) and intermediate-volume (OR 1.32, CI 0.89–1.94; *p* = 0.17) surgeons [10].

In a retrospective review of hospital discharge data of patients undergoing total thyroidectomy between 1998 and 2009 in the Health Care Utilization Project National Inpatient Sample datasets (HCUP-NIS), including 16,954, Adam et al. (ELIII, RGC) found that median annual surgeon volume was 7 cases and that 51% of surgeons performed 1 case per year. Overall, 6% of the patients experienced complications. The likelihood of complication decreased with increasing surgical experience up to 26 cases per year (*p* < 0.01), while 81% of the patients were operated by low-volume surgeons (< 25 thyroidectomies/year) [11]. Patients operated by low- vs. high-volume surgeons were more likely to experience endocrine-related complications (namely hypocalcaemia) (2.3 vs. 1.6%; *p* = 0.01) [11].

Besides surgeon volume, the CATHY study group found that surgeon’s experience, length of practice, and age play a fundamental role in post-thyroidectomy complication rate [14] (ELIII, RGD). In a prospective cross-sectional multicentric study from five academic French hospitals, including 28 surgeons and 3574 thyroid procedures, 20 years or more of practice was associated with increased probability of both recurrent laryngeal nerve palsy (OR 3.06 (1.07 to 8.80); *p* = 0.04) and permanent hypoparathyroidism (OR 7.56 (1.79 to 31.99); *p* = 0.01). Surgeons’ performance had a concave association with their length of experience (*p* = 0.036) and age (*p* = 0.035); surgeons aged 35–50 years had better outcomes than their younger and older colleagues [14].

### Hypoparathyroidism/hypocalcaemia in benign diseases (autoimmune disorders vs. multinodular goiter)

Thomusch et al. (ELIII, RGC) published in 2000 a multivariate analysis of risk factors for postoperative complication over 7266 patients (Table 4) who underwent to thyroidectomy in 45 hospitals for benign disease. In the logistic regression analyses for transient and permanent hypoparathyroidism, extent of resection (transient OR 1.46, CI 1.32–1.63; *p* < 0.0001; permanent OR 1.77, CI 1.52–2.06; *p* < 0.001), recurrent goiter (transient OR 1.82, CI 1.30–2.56; *p* < 0.0001; permanent OR 1.91, CI 1.11–3.27; *p* = 0.018), Graves’ disease (transient OR 2.83, CI 1.93–4.14; *p* < 0.001; permanent OR 2.83; CI 1.58–5.05; *p* < 0.0005), operative volumes (transient OR 0.78; CI 0.65–0.93; *p* = 0.01; permanent OR 1.53; CI 1.08–2.19; *p* = 0.02), and patient gender (transient OR 2.33; CI 1.74–3.13; *p* < 0.0001; permanent OR 2.05, CI 1.29–3.27; *p* = 0.002) were significant risk factors. Patient age also significantly affected the incidence of transient and permanent hypoparathyroidism, although the clinical effect (transient OR 1.01, CI 1.00–1.02; *p* < 0.0001; permanent OR 1.01, CI 1.00–1.03; *p* = 0.02) was limited [5].

**Table 4:** Overview publications regarding volume and outcome in thyroid surgery

Reference	Date of publication	Main outcomes
Sosa [4] et al.	1998	Statewide cross-sectional analysis including 5860 patients. Individual surgeons categorized according to the total 6-year volume (1–9 vs. 10–29 vs. 30–100 vs. > 100 thyroidectomies). Similarly, total hospital volume was categorized in 4 categories (1–99, 100–199, 200–300, > 300). Highest-volume surgeons had the shortest length of stay and the lowest complication rate. Hospital volume had no consistent association with outcomes.
Thomusch [5] et al.	2000	Prospective multicentric study including 7266 patients who underwent thyroid resection for benign diseases along 1 year (01/01/1998–31/12/1998). Hospitals were categorized as low-, intermediate-, and high-volume (< 50, 50–150, and > 150 procedures, respectively). No significant difference was found among hospital groups with regard to post-thyroidectomy morbidity and mortality rate.
Gourin [6] et al.	2010	Statewide cross-sectional analysis including 21,270 patients. Individual surgeons categorized according to the annual volume in low-, intermediate-, and high-volume (≤ 3, 4–24, > 24 thyroidectomies/year). Similarly, hospital volume was categorized in 3 categories (≤ 22, 23–100, > 100). Multiple logistic regression analysis of variables associated with thyroid surgery-specific complications revealed an association with surgeon but not with hospital volume. High-volume surgeons had a lower incidence of laryngeal nerve injury (OR 0.46, *p* < 0.01) and hypocalcaemia (OR 0.49, *p* < 0.01).
Loyo [7] et al.	2013	Nationwide cross-sectional analysis (Nationwide Inpatient Sample) including 871,644 patients. Individual surgeons categorized according to the annual volume in very low-, low-, intermediate-, and high-volume (≤ 3, 4–9, 9–23, and > 24 thyroidectomies/year). Similarly, hospital volume was categorized in 4 categories (≤ 25, 26–42, 43–76, and > 76). Multiple logistic regression analysis of variables associated with thyroid surgery-specific complications demonstrated that recurrent laryngeal nerve palsy and hypocalcemia were significantly less likely for high-volume surgeons (OR 0.71, CI 0.53–0.95, *p* = 0.24 and OR 0.7, CI 0.57–0.88, *p* = 0.002, respectively). After adjusting for surgeons’ volume, hospital volume was not associated with complication rate.
González-Sánchez [8] et al.	2013	Single-institution prospective cohort study including 225 patients and 8 surgeons (2 endocrine surgery specialized with a case load > 40 procedures/year and 6 endocrine non-specialized general surgeons with a case load < 5 procedures/year). Permanent recurrent laryngeal nerve palsy and hypocalcemia were significantly reduced for specialized high-volume surgeons (1/325 vs. 2/46 *p* = 0.04 and 3/130 vs. 3/16 *p* = 0.028, respectively).
Kandil [9] et al.	2013	Nationwide cross-sectional analysis (Health Care Utilization Project National Inpatient Sample datasets - HCUP-NIS) including 46,261 patients. Individual surgeons were categorized according to the volume of procedures performed over the 10-year study period (low-, intermediate-, and high-volume < 10, 10–99, ≥ 100 procedures, respectively). High-volume hospitals were considered those above the 75th percentile with regard to year case load. High-volume surgeons had a significantly lower rate of complications. Hospital volume had an inconsistent and marginal protective effect on postoperative outcomes.
Al-Qurayshi [10] et al.	2016	Nationwide cross-sectional analysis using the National Inpatient Sample datasets, including 77,863 patients. Surgeons were categorized based on the annual case load (low-, intermediate-, and high-volume 1–3, 4–29, ≥ 30 thyroidectomies/year, respectively). Procedures performed by low-volume surgeons were associated with a higher risk of postoperative complications compared with high-volume surgeons (15.8% vs. 7.7%, OR 1.55, CI 1.19–2.03, *p* = 0.01). A surgeon’s expertise (measured by surgical volume of procedures per year) is associated with favorable clinical and financial outcomes.
Adam [11] et al.	2016	Nationwide cross-sectional analysis (Health Care Utilization Project National Inpatient Sample datasets - HCUP-NIS) including 16,954 patients. Surgeons categorized in low-volume (≤ 25 procedures/year) and high-volume (> 25 procedures/year). Patients undergoing thyroidectomy by low-volume surgeon were more likely to have any complication (OR 1.52, CI 1.16–1.97, *p* = 0.002) and a longer hospital stay (+ 12%, *p* = 0.006).
Liang [12] et al.	2016	Nationwide cross-sectional analysis of data of 125,037 patients obtained by the Taiwan Bureau of National Health Insurance and systematic review and meta-analysis of the literature. Surgeons were categorized in low- and high-volume (1–70 and > 70 thyroidectomies/year, respectively) as well as hospitals (1–200 and > 200 thyroidectomies/year, respectively). Patients who received thyroidectomies performed by high-volume hospitals and surgeons had shorter length of stay and lower costs compared with those treated by low-volume hospitals and surgeons.
Nouraei [13] et al.	2017	Nationwide cross-sectional analysis using record of 72,594 patients obtained from the Hospital Episode Statistics (HES) dataset. High-volume surgeons achieve lower complication rates, including lower vocal palsy rates, and length of stay.
Duclos [14] et al.	2012	Prospective cross-sectional multicentric study from five academic French hospitals, including 28 surgeons and 3574 thyroid procedures, 20 years or more of practice was associated with increased probability of both, recurrent laryngeal nerve palsy (odds ratio 3.06 (1.07 to 8.80), *p* = 0.04) and permanent hypoparathyroidism (7.56 (1.79 to 31.99), *p* = 0.01). Surgeons’ performance had a concave association with their length of experience (*p* = 0.036) and age (*p* = 0.035); surgeons aged 35–50 years had better outcomes than their younger and older colleagues.
Mitchell [15] et al.	2008	Single-institution retrospective analysis on 335 thyroid and parathyroid reoperations. Many thyroid and parathyroid reoperations are avoidable. Most originate from low-volume centers. In addition to decreasing complication rates, thyroid and parathyroid surgery performed high-volume centers would decrease the need for patients to undergo reoperations.

Pesce et al. (ELIV, RGD) in an institutional retrospective study compared 68 patients who underwent thyroidectomy for Graves’ disease from 1998 to 2009 with 55 controls randomly selected from the institutional database who underwent total thyroidectomy for benign disease during the same time period. They found that patients with Graves’ disease more likely had a significantly greater number of calcium tablets prescribed upon discharge (1.85 vs. 1.00, CI 1.40–2.46; *p* < 0.001) [20].

In a retrospective, single institution, single surgeon, study, Welch et al. (ELIV, RGD) compared the outcome of total thyroidectomy in 111 patients with Graves’ disease vs. 283 patients operated for non-toxic multinodular goiter between 1996 and 2010. Transient hypocalcaemia was more common in patients with Graves’ disease (72% vs. 60%; *p* < 0.05), but not when matched for thyroid weight [20]. No significant difference was found with regard to other complications (neck hematoma 0% vs. 1%, permanent inferior laryngeal nerve injury 0% vs. 1%; and permanent hypoparathyroidism 1% vs. 0.4%) (*p* = ns) [21].

Hallgrimsson et al. (ELIV, RGD) retrospectively compared a series of 128 patients with Graves’ disease and 81 patients with MNG over a 10-year period (1999–2009). Symptoms of hypocalcaemia were more common in patients with Graves’ disease (*p* < 0.001; OR 95% CI 3.26;1.48–7.14), but the frequency of biochemical hypocalcaemia, postoperative levels of parathyroid hormone (PTH), and treatment with calcium and vitamin D did not differ between groups of patients [22].

Kandil et al. (ELIII, RGC) used the nationwide inpatient sample to identify all patients who underwent total thyroidectomy between 2000 and 2009 and identified 46,261 procedures. In this study, the effects of surgeon volume and hospital characteristics on predicting patient outcomes were analyzed. In addition, univariable and multivariable analyses were used to examine the effects of the indication for surgical care (benign disease vs. thyroid malignancy vs. Graves’ disease) on postoperative outcomes. Patients with Graves’ disease had the highest postoperative complications (17.5%) compared with patients undergoing total thyroidectomy for either benign (13.9%) or malignant (13.2%) thyroid disease (*p* < 0.001). After stratification by surgeon volume, Graves’ disease was found to be a significant predictor of postoperative complications in surgeries performed by low-volume and intermediate-volume surgeons (OR 1.39; 95% CI 1.08–1.79; *p* = 0.01 and OR 1.34; 95% CI 1.06–1.69; *p* = 0.02, respectively). However, Graves’ disease was not a significant predictor of postoperative complications when performed by high-volume surgeons (OR 1.07; 95% CI 0.62–1.83; *p* = 0.81), although hypocalcaemia was not specifically stratified to surgical indications for surgery in this study [9].

In a systematic review and meta-analysis of predictors of post-thyroidectomy hypocalcemia, Edafe et al. (ELIII, RGB), evaluating four studies with a total of 6681 included patients, found that patients with Grave’s disease have a significantly higher risk of transient hypocalcemia when compared with patients without Graves’ disease (OR 1.75, CI 1.34–2.28; *p* < 0.001) [23].

In a retrospective cohort study including 1791 patients who underwent thyroidectomy at a single institution from May 1994 to December 2009, McManus et al. (ELIV, RGD) found that 30 of 311 patients with Hashimoto’s thyroiditis (HT) (9.6%) experienced transient hypoparathyroidism, compared with 75 of 1480 patients without HT (5.1%) (*p* = 0.002). When excluding patients with malignancy, 28 of the 225 patients with HT (12.4%) experienced a postoperative complication, compared with 59 of 1027 patients without HT (5.7%) (*p* = 0.0001) [24].

In a retrospective cross-sectional analysis using the Nationwide Inpatient Sample database from 2006 to 2011, including 215,068 patients who underwent total thyroidectomy (11,205 for Graves’ disease, 110,124 for multinodular goiter, and 93,739 for thyroid malignancy), Graves’ patients had significantly higher rates of hypocalcemia (12.4% vs. 7.3% and 10.3%; *p* < 0.01), hematomas requiring reoperation (0.7% vs. 0.4% and 0.4%; *p* < 0.01), and longer mean hospital stay (2.7 days vs. 2.4 and 2.2 days; *p* < 0.01) compared with multinodular goiter (MNG) and thyroid cancer patients, respectively [24] (ELIII, RGD). In risk-adjusted multivariate logistic regression, Graves’ disease was independently associated with a higher risk of vocal cord paralysis (OR 1.36; CI 1.08–1.69), tracheostomy (OR 1.35; CI 1.1–1.67), postoperative hypocalcemia (OR 1.65; CI 1.54–1.77), and hematoma requiring reoperation (OR 2.79; CI 2.16–3.62) compared with MNG. Graves’ patients treated at high-volume centers (case load > 47 cases per year) experienced lower rates of postoperative hematoma (1.4% vs. 3.1%; *p* < 0.01), hypocalcemia (7.0% vs. 13.9%; *p* < 0.01), tracheostomy (0.2% vs. 1.3%; *p* < 0.01), wound complications (0.0% vs. 0.2%; *p* < 0.01), and major medical complications (1.2% vs. 3.4%; *p* < 0.01) compared with those treated at low-volume hospitals (annual case load ≤ 47 thyroidectomies) [25].

In a prospective multicenter study including 22,011 who underwent thyroid surgery between July 2010 and December 2012 in 68 hospitals of six European countries (Prospective Evaluation of Thyroid Surgery-PETS 2 study), Thomusch et al. (ELIII, RGC) evaluated outcome of 2488 patients who underwent thyroidectomy for autoimmune thyroid disease (including Hashimoto thyroiditis, Graves’ disease, and De Quervain thyroiditis) vs. a control group of 16,467 patients who were operated on for multinodular goiter (MNG). The rates of transient hypocalcemia and definitive hypoparathyroidism were significantly different among the study groups (transient hypocalcemia MNG 12.9% vs. Hashimoto 15.3% vs. Graves’ 20.0% vs. De Quervain 14.0% vs. Riedel 16.7%; *p* < 0.001; definitive hypoparathyroidism MNG 0.9% vs. Hashimoto 1.1% vs. Graves’ 2.8% vs. De Quervain 7.0% vs. Riedel`s thyroiditis 0%; *p* < 0.001) [18]. Surprisingly, Riedel`s thyroiditis had the second highest prevalence of hypocalcemia and no hypoparathyroidism. However, incidence of Riedel`s thyroiditis is so low that this aspect may not be concluded. In logistic regression analysis, surgery for Graves’ disease and Hashimoto’s thyroiditis were found to be independent risk factors for transient hypocalcemia (hazard ratio 2.76 and 8.19, respectively; *p* < 0.001). Graves’ disease was found to be independent risk factor for definitive hypoparathyroidism (hazard ratio 1.57; *p* < 0.001) [19].

### Hypoparathyroidism/hypocalcaemia in benign vs. malignant thyroid diseases

Gourin et al. (ELIII, RGC) in a series of 21,270 surgical procedures performed by 1034 surgeons at 51 hospitals found that the incidence of hypocalcaemia after total thyroidectomy was 10% and was greater for thyroid cancer patients (10.8%) compared with those treated for benign disease (9.3%) (OR 1.28; 95% CI 1.12–1.47; *p* = 0.001) [6].

Loyo et al. (ELIII, RGC) found that the rate of postoperative hypocalcaemia following thyroidectomy was significantly associated with a diagnosis of thyroid cancer (OR 1.30; 95% CI 1.21–1.39; *p* < 0.001) and a concomitant neck dissection (OR 1.95 ;95% CI 1.68–2.26; *p* < 0.001) [7].

Nouraei et al. (ELIII, RGC) recently analyzed national trends, outcomes, and volume-outcome relationships in thyroid surgery in a large cohort of 72,594 patients who underwent elective thyroidectomy in the UK between 2004 and 2012 with 51% hemithyroidectomy and 32% total thyroidectomy for benign (52.5%), benign inflammatory (21%), and malignant (17%) thyroid diseases. In this series, hypocalcaemia was recorded in 1488 patients (2.05% with the highest incidence being observed in patients undergoing thyroidectomy for thyrotoxicosis and thyroiditis (4.34%), followed by surgery for malignant thyroid disease (2.74%)). Moreover, a diagnosis type was an independent risk factor for hypocalcaemia with thyroid malignancy having an OR of 1.59 (CI 1.38–1.79), and benign thyroid conditions having an OR of 1.59 (CI 1.38–1.79). Performing concomitant neck surgery increased the OR of hypocalcaemia to 1.49 (CI 1.20–1.86) [13].

Chadwick et al. (ELIII, RGD) recently analyzed the rate of hypocalcaemia after total bilateral thyroidectomy in the BAETS registry, over 90,000 endocrine procedures in the interval July 2010 to June 2015. It seems apparent that following surgery for Graves’ disease, there is a higher early requirement for calcium supplementation than after a similar extent of thyroid resection for benign disease. By 6 months, however, much of this effect is lost. In contrast, surgery for cancer is significantly more likely to lead to hypocalcaemia, both early and at 6 months, and much of this effect seems to be related to any concomitant central nodal dissection [26].

In a nationwide study based on the data obtained from the South Korea Health Insurance Review and Assessment Review database, data of 192,333 patients who underwent thyroidectomy over a 6-year period were reviewed, 52,707 who underwent thyroidectomy alone and 139,626 who underwent total thyroidectomy plus central neck dissection [27] (ELIII, RGC). The incidence of permanent hypocalcemia was greater in the group of patients who underwent concomitant central neck dissection (5.4% vs. 4.6%, *p* < 0.001). Central neck dissection did not raise the rates of permanent hypocalcaemia in institutes with a low volume of annual cases (< 200), whereas permanent hypocalcaemia was more common in the total thyroidectomy plus central node dissection group than in the total thyroidectomy alone group (3.5% vs 2.9%; *p* = 0.002) in institutes with a large volume of annual cases (≥ 800) [27]. However, overall, complication rate in high-volume centers was significantly lower with respect to low-volume centers, even in the case of the association of central neck dissection [27].

### Propositional summary postoperative hypoparathyroidism and volume

1. Surgeon volume and outcome relationship exists in thyroid surgery with respect to prevalence of hypocalcemia/hypoparathyroidism. A cut-off value of 50 thyroidectomies for a single surgeon per year appears reasonable for identifying a high-volume surgeon.
2. Hospital volume and outcome relationship is less clear than surgeon volume in thyroid surgery with respect to the prevalence of hypocalcemia/hypoparathyroidism. However, a cut-off value of 100 thyroidectomies for a single center per year appears reasonable for identifying an adequate high-volume unit.
3. Surgery for thyroid cancer is a predictor of increased risk of hypocalcemia/hypoparathyroidism in the pooled cohort of surgical volume, but the rate is lower for high-volume surgeons.
4. Surgery for AITD is a predictor of increased risk of hypocalcemia/hypoparathyroidism in the pooled cohort of hospital volume, but the rate is lower for high-volume units.
5. Thyroid surgery for thyroid malignancy performed by low-volume surgeons is associated with an increased risk of recurrence. Reoperative surgery is associated with an increased risk of hypocalcemia/hypoparathyroidism.
6. Current evidence suggests that total thyroidectomy for thyroid cancer or AITD should be undertaken by high-volume surgeons who have lower rates of hypocalcemia/hypoparathyroidism than low-volume surgeons.

### Postoperative hemorrhage

Postoperative hemorrhage represents the one emergency event in thyroid surgery. It is potentially life-threatening, can lead to irrevocable hypoxic brain damage, and may further result in recurrent nerve palsy, hypoparathyroidism, or wound infection. Despite identification of risk factors, from the surgeon`s perspective, the event of bleeding remains largely unpredictable. Moreover, continuous efforts to refine the technique of thyroid dissection, advances in technology, and application of coagulation-assisted devices as well as local hemostyptica failed to improve rates of postoperative hemorrhage. Only indirectly, a minor improvement can be assumed when considering that the extent of resection has considerably increased in the past decades, and rates of bleeding remain stable. Logistic structures of postoperative care are of paramount importance for the outcome in the event of hemorrhage following thyroid surgery. Thus, due to prevalent surgically unswayable risks, bleeding rate is a feature that while representing a quality indicator, must also be assessed in its own outcome quality.

Incidence of postoperative hemorrhage over all types of thyroid disease and surgery spans broadly from 0.3 to 3.5%. Moreover, the boundaries for defining surgeon and hospital volume categories are mixed. Low volume starts from 1 to 9 vs. < 50 vs. < 100 per annum vs. overall experience, while high volume may be defined from 20 to > 300 thyroid surgeries per annum vs. overall experience. Thus, comparison of already heterogenous studies is compromised. Publications specifically addressing postoperative hemorrhage and outcome volume are scarce, and in some instances are included in studies that look at all over outcome-volume aspects. These are predominantly of retrospective design and generally exhibit low evidence levels III and recommendation grades C–D.

### Postoperative hemorrhage in thyroid surgery and surgeon volume

A clear number of studies investigating postoperative hemorrhage in thyroid surgery did not identify surgeon volume to significantly influence bleeding rate. In these, bleeding rates range from 0.3 to 4.4% according to surgeon category. Equal distribution of postoperative hemorrhage between surgeon categories and experience is demonstrated by Bergamaschi et al. [28] (ELIII, RGD), Wang et al. [29] (ELIII, RGD), Loyo et al. [7], Perera et al. [30] (ELIII, RGD), Lorenz et al. [31] (ELIII, RGC), Gurrado et al. [32] (ELIII, RGD), and Meltzer et al. [33] (ELII, RGC) (Table 5). None of these studies specifies outcomes or consequences in the event of postoperative hemorrhage.

**Table 5:** Surgeon volume and outcome for post-thyroidectomy hemorrhage

Author, year	Patients/procedures; disease (*n*)	Revision for hematoma/conservative (*n*/*n*)	Bleeding rate (%) overall/surg category	Outcome	Surgeon volume criteria	EL/RG	Comment	Suggested surgeon volume
Without impact surgeon volume								
Bergamaschi [28] et al., 1998	1192/all types; benign and cancer	19/10	1.6	No further impairment	Low < 50 intermediate 50–100 high > 100	III/C	Equal distribution surg cat	No signif. diff. surg. vol. no minimum number
Sosa [34] et al., 2008	1199 pat. TT+PTX in pat. < 17years	NA	3.4%	Overall compl other 9.5% pediatric 11.0% high 5.6%, ns	Other pediatric high	IV/D		Low vol. surg powerful predictor worse outcome
Tuggle [35] et al., 2011	6762 thyroidectomy same day discharge					IV/D	Same day disch. more often high vol surg (*p* > 0.001)	No minimum number
Loyo [7] et al., 2013	871,644	NA	NA		Proc./annum ≤ 3/a very low 4–9/a low 9–23 intermediate > 23/a high	III/C		No minimum number
Perera [30] et al., 2015	205/HT+TT	9/205	4.4	Hemorrhage: A: 5/114 B: 4/91 (*p* = 0.78)	A: trainee B: surgeon	III/C		No diff. regarding hemorrhage for surg cat
Liang [12] et al., 2015	125,037 pat.	NA	NA		1–70/a low > 70/a high specific postop complic. intermediate surg OR 0.63 *p* = 0.86 high surg OR 0.63 *p* < 0.001	III/C	All over LOS 0.2 days (6.3%) *p* < 0.001; in-hospital mortality equal *p* = 1.000 § LOS high 1.2–3.2 vs. low 1.7–3.5 days; in-hospital mortality favorable high OR 5.99 (95% CI 1.98–18.12; *p* = 0.002); all over postop surg. complication OR 0.72 *p* < 0.001	Postop complications inversely correlated with surgeon volume; hemorrhage not specified
Gurrado [32] et al., 2016	8908/TT	NA	NA	Overall morbidity (22.3%) sign. ass. with A: OR 1.48 (95% CI 1.12–1–96; *p* = 0.005) B vs. A: 29.5%/22.3% and C: 21.3% hemorrhage: A: 53/7092 (0.7%) B: 1/261 (0.4%) C: 6/1555 (0.4%) OR B+C vs. A: 0.51 (0.23–1.13) *p* = 0.10	A: resident/cons. B: consultant C: resident/res.	III/C		No diff. regarding hemorrhage for surg cat
Meltzer [33] et al., 2016	3135/HT vs. TT/benign	NA	NA	All over complic lower in HVS: 5.7% vs. 7.5% *p* < 0.05 hemorrhage TT: LVS: 0.4/1779 HVS: 0.7/1779 (*p* = 0.25) hemorrhage HT: LVS: 0.3/1356 HVS: 0.6/1356 (*p* = 0.25)	LVS: ≤ 20/proc/a IVS: 21–40/proc/a HVS: > 4/proc/a	IV/C		No diff. regarding hemorrhage for surg cat
With impact surgeon volume								
Sosa [4] et al., 1998	5860 pat. all types		Mean 1.5%		Total proc./6 years A: 1–9 = 2.0% B: 10–29 = 1.6% C: 30–100 = 1.2% D: > 100 = 1.4%	III/C		Sign. assoc. improved pat. outcome with increased surg vol (D 1/3 complic vs. A)
Mishra [36] et al., 1999	232/TT; benign and cancer	6/NA	2.6/1.6 vs. 3.8	Not specified	Cumulative proc. 1–120 in 1.6–8 years	III/C	Consultant vs. trainee 127/105	Signif. diff surg vol *p* > 0.05 diff. no minimum number
Stavrakis [37] et al., 2007	10,009/NA	NA	NA *2.77%	LOS prolonged; costs increased LOS 0.06 days/surg cat or decreased cost $365^*^	Proc./annum A: 1–3 B: 4–8 C: 9–19 D: 20–50 E: 51–99 F: ≥ 100	IV/C	All over compl. signif. higher A vs. F *p* < 0.02 inverse correlation surg cat to hemorrhage; hemorrhage single parameter influenced by surg vol; no hospital vol effect on compl. seen	Signif. diff surg vol. *p* < 0.10 no minimum number
Sosa [38] et al., 2008	22,848/all types; benign and cancer/age > 65 years/≥ 80 years	NA	NA	High vol surg sign. lower all over endocr. compl. 4.5% ≥ 80 years vs. low vol surg (13%); higher costs; LOS 2.3 days vs. 4.8 days; in-hospital mortality	Very low: 1–9/a low: 10–29 intermediate: 30–100/a high: > 100/a	III/C	All over endocrine complic. signif. higher in low surg vol for pat > 65 years, more so pat ≥ 80 years	Signif. diff surg vol* no minimum number
Tuggle [39] et al., 2008	607/TT/PTX/age ≤ 17 years	NA	NA	High vol surg improved outcome: fewest compli general/endocrine high:8.7%/5.6% ped.: 13.4%/11% other: 13.2%/9.5%	Pediatric (2 proc/a) other (M 7 proc/a) high (M 72 proc/a)	IV/C		Signif. diff surg vol.* *p* < 0.10 no minimum number
Boudourakis [40] et al., 2009	Cross-sectional in patient database various surgical proc 1999–2005: TT	NA	NA	High vs. low: mortality 1999: 2.5%/7.1% mortality 2005: 4.9%/7.9% general compl 1999: 2.5%/7.1% 2005: 4.9%/7.9% LOS 1999: 1.4d/2.4 LOS 2005: 1.3 days/3.9 days OR mortality: low vs. high 1999: 1.8 2005: 1.4	Low:9/a medium high:30/a	III/C	Increase proportion high vol surg 23% in 6 years	Signif. diff surg vol.* *p* < 0.001 no minimum number
Kandil [9] et al., 2013	46,261 proc	NA	NA	*Tracheostomy low 0.05% intermediate 0.02% high 0% *in-hospital mortality low 0.2% intermediate 0.03% high 0.02%	Proc/10 years < 10 low 10–99 intermediate ≥ 100 high	III/C	Low 2.3% intermediate 1.6% high 1.0%	Diff. surg vol influences hematoma rate
Adkisson [41] et al., 2014	1249/HT+TT/DTC ≥ 1 cm	3/359	0.8	High vol surg signif less all over compl in all categories hemorrhage: HVS 1/175 (0.4%) LVS 2/84 (2%)	10/a 20/a 25/a (< 30 LVS) 30/a (≥ 30 HVS) 50/a 100/a	III/C		Diff surg vol *p* < 0.58 no minimum number
Hauch [42] et al., 2014	62,722/HT vs. TT/benign	TT vs. HT: 1.54% vs. 1.24% (*p* = 0.0027)	NA	LVS higher OR overall compl vs. HVS: 1.53 (95% CI 1.12, 2.11; *p* = 0,0083 LVS: 24.1% complic IVS: 18.8% HVS: 14.5% hemorrhage: LVS: 0.274/18,954 IVS: 0.103/16,797 HVS: 0.000/1799	LVS: <10 proc/a IVS >10 < 99 proc/a HVS: > 99 proc/a	III/C	Hospital vol associated with gen. complic (*p* = 0.0046)	Signif. diff surg vol.* *p* < 0.0001 no minimum number
Adam [11] et al., 2017	16,954/TT/ benign and cancer	254	1.5	All over complic 6% LVS vs. HVS OR: 1.51 (*p* = 0.002) LOS + 12%, *p* = 0.006) hemorrhage: LVS: 1.6% HVS: 1.0% (*p* = 0.006)	LVS: ≤ 25/proc/a HVS	III/C	Median vol all surg: 7 linear decline all over complic. with no./proc performed/a	Sign. improved outcome* at ≥ 25/proc/a

*DTC* differentiated thyroid cancer, *HT* hemithyroidectomy, *LOS* length of hospital stay, *TT* total and near-total thyroidectomy, *proc. procedure* thyroid/±parathyroid resection, *NA* not assessed or not provided

*All over postoperative complication without specifically detailing hemorrhage

^§^Meta-analysis data additionally provided

Contrarily, a larger number of studies directly describe or indirectly imply significant associations in the selected surgeon volume categories with postoperative hemorrhage such as Sosa et al. [4, 34, 38], (ELIII, RGC) (ELIII, RGD) (ELIII, RGD), Wang et al. [29] (ELIII, RGD), Mishra et al. [36] (ELIII, RGD), Stavrakis et al. [37] (ELIII, RGD), Tuggle et al. [35] (ELIII, RGC), Boudourakis et al. [40] (ELIII, RGD), Tuggle et al. [43] (ELIII, RGC), Promberger et al. [41] (ELIII, RGC), Campbell et al. [42], (ELIII, RGD), Kandil et al. [9], Hauch et al. [39], (ELIII, RGC), and Liang et al. [12] (Table 6). On the other hand, Adam et al. [11] described a lower bleeding rate for high-volume surgeons of 1% vs. 1.6% (*p* = 0.006), likewise Adkisson et al. [44] (ELIII, RGD) with 0.4% high-volume vs. 2% (*p* = 0.07) for low-volume surgeons. Kandil et al. [9] (ELII, RGD) found bleeding rates for three surgeon volume groups, high, intermediate, and low to be 0.02%, 1.6%, and 1%, respectively (*p* = 0.001). Stavrakis et al. [37] show an inverse, however, nonlinear correlation of complication rate with surgeon volume for postoperative hemorrhage at *p* < 0.05. Mishra et al. [36] demonstrate a significant difference for bleeding of 1.6% high- vs. 3.8% (*p* < 0.005) for low-volume surgeons. Dehal et al. [47] describe a graduated bleeding rate for surgeon volume categories high, intermediate, and low of 0.9%, 1.4%, and 2.1% at an over all rate of 1.5%. In these, bleeding rates range from 0.8 to 3.8%. Stavrakis et al. are the only to state that postoperative hemorrhage is the single parameter influenced by surgeon volume. Only few studies further assess outcome aspects in the event of postoperative hemorrhage like all over higher complication rates in lower volume categories and/or prolonged length of stay and/or increase of costs [34, 37, 38, 43]. Relevant quality parameters associated with poorer outcome in low-volume category surgeons are in-hospital mortality [4, 9, 11, 12, 39, 40, 44] and tracheostomy rate [9]. Only the study by Promberger et aI. [41] specifically assessed important complications linked with postoperative hemorrhage: 3 (0.6%) deaths and 9 emergency tracheostomies (1.7%).

**Table 6:** Hospital volume and outcome for post-thyroidectomy hemorrhage

Author, year	Patients/departments/procedures; disease (*n*)	Revision for hematoma/conservative (*n*/*n*) all over bleeding rate	Bleeding rate (%) HV	Outcome	EL/RG	Hospital volume criteria	Suggested hospital volume
Sosa [4] et al., 1998	5860	NA	NA	NA	III/C	Thyroidectomy/year 1–99 100–199 200–300 > 300	Hosp vol no consistent correl with outcome
Sosa3 [37] et al., 2008	1199 pat. TT+PTX in pat. < 17 years	NA	Overall compl low 11.1% high 7.7% *p* = 0.05	NA	IV/D	Low high	No minimum number
Godballe [44] et al., 2009	5490 12 departments (3 with > 100/a) performed 63% of study	230/5422	Overall 4.2 median 4.7 (1.9–14.3%) 4.6 only benign histology no minimum number	NA	IV/D	Not detailed	No minimum number
Weiss [39] et al., 2010	150,012	1.34%	LV 2.3% IV 1.6% HV 1%		III/C	HV decreased risk; mortality in bleeding increased	No minimum number
Tuggle [35]et al., 2011	6762 thyroidectomy same day discharge	NA	NA	NA	IV/D	Same day disch. more often high vol hosp. (*p* > 0.001)	No minimum number
Loyo [7] et al., 2013	871,644 pat.	NA	NA	All over postop surg. complication OR 0.83 *p* = 0.12 specific postop complic. high vol hosp OR 0.72 *p* = 0.031	III/C	Proc./annum ≤ 25/a very low 26–42/a low 43–76 intermediate > 76/a high	No minimum number
Kandil [9]et al., 2013	46,261 proc	NA	NA	NA	IV/D	Total thyroidectomy > 75 percentile TT/year	Hosp vol inconsistent and marginal effect all over outcome
Dehal [45] et al., 2014	147,344	1.5%				HV no effect; mortality in bleeding increased	No minimum number
Hauch [42] et al., 2014	62,722	1.2%	LV 1.54% HV 1.24%		III/C	NA	No minimum number
Liang [12] et al., 2016		NA	NA	NA	II/C	1–200 proc/a low > 200 proc/a high	No minimum number
Maneck [46] et al., 2017	66,902	NA	I 1.5% II 1.8% III 1.8% IV 1.9% V 1.9%	NA	IV/D	I 1–49 II 50–89 III 90–180 IV 181–384 V 385–2052	No minimum number
Thomusch [19] et al., 2018	22,011, benign and AIT	NA	NA	NA	III/C	NA	No minimum number
Bartsch [43] et al., 2019	12,888 benign goiter	180	1.4% benign goiter vs. 1.7 Graves‘		III/C	All over rate, no volume correlation prov.; hosp. vol. range < 50 proc/a-> 300 proc/a	No minimum number

Although all studies refrain from stating a minimum number for certain procedures, a specifically surgeon-dependent raised bleeding rate by 7-fold is described by Promberger et al. in an exclusively high-volume surgeon group with > 300 thyroid procedures in all [41]. The majority of studies state a significantly to clearly improved (*p* < 0.0001–*p* < 0.58) outcome in favor for the high-volume category respectably defined or an inverse correlation of complications with surgeon volume, while only Adam et al. [11] identify improved outcome at ≥ 25 procedure/year/surgeon (Table 6).

### Postoperative hemorrhage in thyroid surgery and hospital volume

A smaller number of studies describe correlation of bleeding rates with hospital volume, directly or indirectly in alliance with all over complications. Hospital volume categories range from 1–99, < 25, and < 50 thyroid procedures/year to > 76, > 200, and > 300 thyroid procedures/year. Bleeding rates herein range for most from 1.4 to 4.7% [4, 7, 9, 12, 19, 34, 43, 48, 49] (ELIII, RGD) [50] (ELII, RGC) (Table 6). Godballe et al. [45] describe a wide range of bleeding rates between centers from 1.9 to 14.3% at a median rate of 4.2%. Few of the available studies clearly identified hospital volume to be positively associated with postoperative hemorrhage while all over complications demonstrate differences in the respective hospital categories. Hauch et al. [39] show a significantly improved postoperative hemorrhage rate for high-volume centers of 1.24% vs. 1.54% (*p* = 0.0027). Weiss et al. [45] (ELIII, RGD) show an improved bleeding rate for high-volume hospitals (OR 0.71) at all over bleeding rate of 1.25%, while patients that incur postoperative hemorrhage have higher mortality than patients without (1.34% vs. 0.32%; *p* < 0.001). In some studies, postoperative hemorrhage is thereby included in the outcome analyses. High-volume centers performing same day discharge for thyroid procedures are likely to bias results regarding postoperative hemorrhage due to preselection of patients. Studies that demonstrate improved outcomes for high-volume centers vs. low-volume are Sosa et al. [37] (7.7% vs. 11.1%; *p* = 0.05), Wang et al. [29] (5.6% vs. 8.7%), and Loyo et al. [6] (OR 0.72; *p* = 0.031); Mitchell et al. [15] state hospital volume to be inversely correlated with perioperative complications (bleeding rate 0.597%). Pieracci et al. [51] describe a linear association of increasing volume with decreasing overall complications (bleeding *p* = 0.01). Other studies show inconsistent or marginal effects of hospital volume on outcome [12, 19, 38, 40, 45, 47, 48, 50] (see Table 6).

### Risk factors, time, and outcome of postoperative hemorrhage in thyroid surgery

Regarding postoperative hemorrhage in thyroid surgery, most studies identify auxiliary risk factors besides surgeon and hospital volume (Table 7). Patient-related risk factors mostly affirmed are age, gender, type of thyroid disease, and coagulation medication. Similar importance is extent of resection, realization of lymph node dissection, placement of drains, and duration of surgery. Studies concerned with time frames of postoperative hemorrhage show that the majority of bleedings occur within 6 hours from the thyroid procedure (43–81%); however, considerate bleeding develops after 24 hours (17–39%), at 48 hours (2.4–25%) or even as late as 7, 9, and 20 days postoperatively. Outcome in the event of postoperative hemorrhage is scantly investigated, and describes blood transfusion rate of 0.1–7%, recurrent laryngeal nerve palsy 5.1–16.7%, tracheostomy 1.7–16.7%, and mortality of 0.5–1.34%.

**Table 7:** Risk factors and outcome bleeding

Risk factor		Outcome	
Age	> 60 years > 45 years	Transfusion	0.1–7 vs. 0
Gender	Male > > female	RLNP	5.1–7.1 vs. 4.4–4.7
Thyroid disease	GD > > thyroiditis, CA	Tracheostomy	1.7–16.7 vs. 0.1
Type surgery	Redo > primary	Mortality	0.5–1.34 vs. 0.01
Lymph node dissection	TT > > H	-	-
Duration surgery	++	-	-
Coagulation/medication	++/+	-	-

### Propositional summary postoperative hemorrhage and volume

1. Correlation of surgeon volume with bleeding rate is conflicting, and albeit high-volume surgeons generally perform better. No evidence exists for a minimal number of procedures. Bleeding rate < 2% at 50 annual procedures performed appears reasonable. Other studies showed inconsistent or marginal effects of hospital volume on outcome [5, 18, 37–39, 43, 47, 48] (see Table 6).
2. Hospital volume and bleeding rate is less clear than surgeon volume in thyroid surgery. However, a cut-off value of 100 annual thyroidectomies for a single center per year appears reasonable for identifying an adequate high-volume unit.
3. Outcome relationship for the event of bleeding in regard to the specific complications thereof and with surgeon/hospital volume is severely underreported.
4. Outcome quality of bleeding is expected to correlate more with hospital volume than with surgeon volume by structural logistic impact.
5. Patients` risk factors of bleeding are mainly uninfluenced by surgeon and hospital volume.

### Volume and wound complications

Few authors have specifically investigated the influence of volume on the prevalence of wound infection, hematoma, and seroma (Table 8). Neck hematoma has been found to be inversely related to surgeon volume. However, most of the authors agree that wound seroma is not influenced by the number of thyroid procedures performed. It is not clear whether the prevalence of wound infection is related to surgeon volume.

**Table 8:** Volume and wound complications

Author	Year	Country	N	Volume (thyroid procedures/year)	Wound infection		Hematoma/bleeding		Seroma		Wound dehiscence	
					%	*p*	%	*p*	%	*p*	%	p
Thomusch [19] et al.	2000	Germany	7266	Low (< 50)	0.8	NS	NA	NA	NA	NA	0	NS
				Intermediate (50–150)	1.1		NA		NA		0.2	
				High (> 150)	0.4		NA		NA		0.1	
Hauch [42] et al.	2014	USA	62,722	Low (< 10)	0.005	NA	1.727	< 0.0001	0.046	NA	NA	NA
				Intermediate (11–99)	0		1.147		0.011		NA	
				High (> 99)	0		0.549		0		NA	
Kandil [9]et al.	2013	USA	46,261	Low < 10	0.4	< 0.0001	2.3	< 0.0001	0.1	0.22	NA	NA
				Intermediate (10–99)	0.1		1.6		0.02			
				High ≥ 100	0.1		1		0.02			
Meltzer [33] et al.	2016	USA	3135	Low (≤ 20)	1	< 0.05	0.4	0.25	0.6	0.58	0.3	0.40
				High (> 40)	0.3		0.7		0.8		0.4	
Adam [11] et al.	2017	USA	16,954	Low (≤ 25)	1.1	0.05	1.6	0.006	NA	NA	NA	NA
				High (> 25)	0.7		1					

Prevalence of wound complications after thyroid surgery is low, rating from 0.3 to 1.7%. In an Italian retrospective multicentric review of 14,000 patients, Rosato et al. [52] (ELIII, RGD) found that wound infection occurred after 0.3% of all operations, accounting for 2.0% of all the complications. Half of the surgeons applied antibiotic prophylaxis and 17% antibiotic therapy; 33% did not apply any prophylaxis or therapy. Despite these differences, the incidence of infections was not different between these groups.

Bergenfelz et al. [53] (ELIII, RGC) found a 1.6% of wound infection which was associated with lymph node dissection (5% vs. 1.2%), operation due to re-bleeding (5.3% vs. 1.5%) (OR 3.394; 95% CI 1.1568–9.9581; *p* = 0.0261), and decreased in patients operated to exclude malignancy 1.1% (OR 0.382; 95% CI 0.1711–0.8520) as well as in patients operated due to malignancy without lymph node dissection (OR 0.326; 95% CI 0.1162–0.9127; *p* = 0.0329).

In another Swedish multicentric study of 1157 patients operated for Graves’ disease, Hallgrimson et al. [22] found 1% of wound infection which was a risk factor for vitamin D treatment at discharge.

A cross-sectional analysis performed by Hauch et al. [39] using the American Nationwide Inpatient Sample (NIS) database identified 62,722 thyroid procedures for benign disease (57.9% were total thyroidectomies). Although low-volume surgeons were more likely to have overall postoperative complications, wound complication (not specified) was not different between low- (< 10 thyroid surgeries performed), intermediate- (11–99), and high-volume (> 99 thyroid procedures) surgeons. Neck hematoma was inversely related to surgeon volume: low volume (1.72%), intermediate (1.15%), and high (0.55%) *p* < 0.0001. No differences were seen between groups regarding neck seroma.

Using the same American database, Adam et al. [11] found more prevalence of non-specified wound complications in low- (1.1%) vs. high- (0.7%) volume surgeons but without reaching statistical significance (*p* = 0.05). Bleeding, however, was more frequent if the patient was operated by a low-volume surgeon than by a more experienced one (1.6 % vs. 1%; *p* = 0.006).

In the same way, in a German prospective multicenter study assessing complications after thyroid surgery for benign goiter, Thomusch et al. [5] did not find differences in wound infection and wound dehiscence between high- (> 150), intermediate- (50–150), and low- (< 50) volume surgeons.

On the other hand, other authors did find an inverse relationship between surgeon volume and wound infection:

Meltzer et al. [33] assessed complications including surgical site infection, deep incisional surgical site infection, wound disruption, neck swelling, and seroma. Lower rate of site infection was found in high-volume surgeons (0.3%) than in low-volume surgeons (1%) after applying a propensity matching score. Likewise, Kandil et al. [9] found significantly more prevalence of wound infection and neck hematoma in low-volume surgeons than for intermediate- and high-volume surgeons but no substantial differences regarding neck seroma.

### Propositional summary postoperative wound complications and volume

1. The prevalence of postoperative neck hematoma has been found to be inversely correlated to surgeon volume. A cut-off value of thyroid procedures per surgeons has not been established.
2. Seroma is not influenced by the number of thyroid procedures performed.
3. Patients operated on by low-volume surgeons seem to be more prone to wound infection in all the studies, although only two of them reached statistical significance. This may be due to the low prevalence of wound infection and retrospective nature of the studies.
4. Current evidence cannot suggest any strong impact of surgeon volume on wound complications.

### Surgeon volume, extension of surgery, recurrence, and mortality in papillary thyroid cancer

Surgery is the most important treatment modality for papillary thyroid cancer, and the relationship between type of operation and clinical outcomes has been studied extensively. Surgeon-related factors (annual operation volume, years of experience) also play an important role in short- and long-term clinical outcomes of papillary thyroid cancer (PTC) (Table 9).

**Table 9:** Recent studies assessing the relationship between surgeon volume and results for thyroid cancer procedures

Author	Year	Type of study	N	Population characteristics	Main conclusions
Kim [54] et al.	2018	Unicentric	1103	N1b PTC	1.HVS associated with low structural recurrence. 2.Distant metastasis and disease-specific mortality not affected.
Papaleon-tiou [55] et al.	2017	Survey	269^a^	Referring physicians	Those treating > 10 thyroid cancer patients/year select HVS for advanced PTC and/or frail patients.
Youngwirth [56] et al.	2016	Registry^b^	31,129	PTC with TT	1.Both micro- and macroscopically positive margins associated with compromised survival. 2.Reception of surgery at a high-volume facility was protective.
Oltmann [57] et al.	2014	Unicentric	45	TT vs. CT followed by RAI ablation	If a staged operation for cancer is necessary, surgeon volume may impact the completeness of resection
Adkisson [44] et al.	2014	Unicentric^c^	362	DTC ≥ 10 mm	1.Surgeons ≥ 30 TT/year more likely to TT and initial resection more complete. 2.For patients with advanced stage disease, a threshold of ≥ 50 TT/year needed for improvements in I^123^ uptake
Yap [58] et al.	2013	Unicentric	651	TT for DTC TxNxM0 > 3000 MBq ^131^I	1. Clear relationship between volume and size of thyroid remnant. 2. Specialist surgeons: smallest remnant
Schneider [59] et al.	2013	Unicentric	223	TT for DTC+RAI ablation	Remnant uptakes of HVS (≥ 20 TT/year) significantly smaller than LVS.
Gourin [6] et al.	2010	Registry^d^	21,270	Thyroidectomy	Thyroid cancer surgery was less likely to be performed by high-volume surgeons despite an increase in surgical cases
Mitchell [15] et al.	2008	Unicentric	134	Reoperations for DTC	The majority of avoidable thyroid reoperations came from LVC.

*PTC* papillary thyroid cancer; *HVS* high-volume surgeon; *LVS* low-volume surgeon; *TT* total thyroidectomy; *CT* completion thyroidectomy; *RAI* radioactive iodine; *DTC* differentiated thyroid cancer; *LVC* low-volume centers

^a^Members of the Endocrine Society, American College of Physicians and American Academy of Family Practice

^b^The National Cancer Data Base (USA)

^c^University of Pittsburgh Medical Center (USA)

^d^Maryland Health Service Cost Review Commission (USA)

There is a compelling amount of data about the inverse relationship between surgeon volume and postoperative complications after thyroid surgery. Most studies, however, focus on short-term complications rather than the more important long-term oncological outcomes such as structural recurrence, subsequent distant metastasis, and cancer-specific mortality of PTC. As a result, there is a lesser amount of research on the most of the published data that are only for patient populations in the USA and Europe [12].

On the other hand, consistent and robust studies have found associations between surgeon factors and cancer-specific mortality or tumor recurrence for many other cancer types. The relationship between surgeon-related factors and long-term oncological outcomes in PTC received less attention. This issue is critically important, given that adequately performed thyroidectomy is the cornerstone of long-term disease-free survival and furthermore, patients with advanced PTC have a high likelihood of poor prognosis.

### Volume, complexity, and cancer

Several works by Sosa et al. [4, 34, 38], a pioneer in thyroidectomy outcome research, emphasize that surgeon volume is much more important than hospital volume. Interestingly, in a cross-sectional analysis where 21,270 thyroid surgical procedures performed in Maryland (USA) were analyzed, thyroid cancer surgery was less likely to be performed by high-volume surgeons despite an increase in surgical cases. Further investigation is needed to identify factors contributing to this trend [4, 6, 29].

### Completeness of resection

Completeness of resection after total thyroidectomy for papillary thyroid cancer is a crucial factor for biochemical, structural recurrence, and, in some studies, even survival. In a pioneer study to examine margin status after total thyroidectomy for PTC in a nationwide database (National Cancer Database of USA), 31,129 patients who had undergone total thyroidectomy for cancer were analyzed focusing on the relationship between completeness of resection and recurrence and survival. After multivariable adjustment, both microscopically and macroscopically positive margins were associated with compromised survival. Of particular interest is that reception of surgery at a high-volume facility (OR 0.72; *p* = 0.01) emerged as a protective for cancer-related death [56] (ELIII, RGD).

At least two studies identified, in the US health system, several vulnerable patient populations with a higher risk of incomplete resection after thyroidectomy for PTC as low-income patients treated low volume and community hospitals are less likely to undergo total thyroidectomy. These differences in practice patterns likely reflect disparities in access to health care, medication, and comprehensive cancer centers [56, 60] (ELIII, RGD) (ELIII, RGC).

Radioiodine (RAI) remnant uptake has been used as a surrogate parameter to assess the completion of resection and thus seems useful to assess the completeness of a total thyroidectomy. Remnant uptake is a useful postoperative oncologic quality indicator that can predict a patient’s risk of disease recurrence and indicate the completeness of resection [59] (ELIII, RGC). Oltmann et al. [57] (ELIII, RGD) observed that completion thyroidectomy carried out by high-volume surgeons (≥ 30 procedures/year) was associated with much lower remnant uptake at first RAI treatment (0.06 vs. 0.22% for low-volume surgeons; *p* = 0.04) [57] (ELIII, RGD). Yap et al. [58] (ELIII, RGD) reported a significant relationship between the number of thyroidectomies and total body uptake of ^131^I in pre-ablative scans. They demonstrated the incompleteness of tumor resection by RAI uptake and concluded that low surgeon volume was associated with incompleteness of tumor resection, although there was no analysis of tumor recurrence [58].

A single study combined several indicators as initial extent of the operation together with three markers of complete resection including uptake on I^123^ prescan, thyrotropin-stimulated thyroglobulin levels, and I^131^ dose administered to assess the adequacy of thyroidectomy for differentiated thyroid cancer and relate them to surgeon volume. They conclude that surgeons who perform ≥ 30 thyroidectomies/year are more likely to undertake the appropriate initial operation and have more complete initial resection for differentiated thyroid cancer patients. Surgeon volume appeared in this study an essential consideration in optimizing outcomes for differentiated thyroid cancer patients, and even higher thresholds (≥ 50 thyroidectomies/year) may be necessary for patients with advanced disease [44].

### Recurrence

In a retrospective recent study, Kim et al. [54] (ELIII, RGD) analyzed 1103 patients operated on for papillary thyroid cancer with N1b extension. They compared the results from low-volume surgeons (< 100 procedures/year) with high-volume surgeons and stratified them further into more and less experienced subgroups. After 150 months of follow-up, the recurrence-free survival obtained by the high-volume and more experienced surgeons was statistically superior to the remaining groups of surgeons with high- or low-volume and less or more experience. Treatment by a high-volume surgeon was associated with half (14.7% vs. 27%) structural recurrence rate in patients with N1b PTC. This association was maintained after adjustment for age, sex, and conventional risk factors for recurrence of thyroid cancer. High-volume surgeons had a significantly lower rate of resection margin positivity and stimulated thyroglobulin (Tg) level above 10 ng/ml at the time of first radioiodine treatment, representing the completeness of surgical resection. Neither distant metastasis nor cancer-specific mortality was modified by surgeon volume. The study concluded that by far the best combination was provided by experienced surgeons with high volume. Also, this imaginative study emphasizes that low volume seems to cancel the potential advantages of more experience and vice versa [54].

Several other studies have shown the importance of surgical volume in reducing rates of recurrence and complications for thyroid cancer patients undergoing total thyroidectomy [36, 60]. Surgeons who perform > 20 thyroid surgeries a year have lower permanent complication rates and leave less remnant thyroid tissue behind to treat with RAI [59]. The analysis of data from the National Cancer Database showed that having total thyroidectomy performed at an institution that performed less than 12 thyroidectomies a year was associated with compromised survival [56].

Also, several studies have shown that patients operated on by specialized endocrine surgeons with > 10-year experience and/or at institutions with a focus on thyroid cancer have unsurprisingly less remnant thyroid tissue to ablate.

### Reoperations

Mitchell et al. [15] analyzed the relationship between hospital volume and endocrine avoidable operations and demonstrated that operations for thyroid cancer led to avoidable reoperations more frequently if performed at low-volume centers, mainly compromised by a mix of “judgement errors” and “technical errors”. In this retrospective analysis of reoperated patients, it appears obvious that the avoidable operations are a result not only of technical errors, easily attributable to limited exposure and practice, but also to “judgement errors” stemming from a lack of surgical knowledge in low-volume centers [15].

### Propositional summary extension of surgery, recurrence, and mortality in papillary thyroid cancer and volume

1. Low-volume surgeons perform less complete operations for thyroid cancer.
2. Patients with PTC operated on by low-volume surgeons or low-volume centers suffer more recurrences and have to be reoperated more often.
3. No direct relationship between volume and survival of patients with PTC/DTC has been clearly demonstrated.
4. A surgeon performing less than 25 total thyroidectomies per year can be considered a low-volume thyroid surgeon.

## Discussion

The working group in volume outcome following thyroid surgery aimed to evaluate the impact of surgeon and hospital volumes on outcome of thyroid surgery, based on a review of the existing literature. The analysis of the literature confirmed that surgeon’s volume and experience play a significant role in the rate of post-thyroidectomy complications. The role of hospital volume, especially when adjusted for surgeon volume, is more limited or inconsistent [5, 9, 36].

This underlines once more, if still necessary, the need of an appropriate training and subspecialization of surgeons dealing with thyroid surgery. Surgical skill and experience are particularly necessary when dealing with difficult cases, such as autoimmune thyroid disorders (including Graves’ disease and Hashimoto’s thyroiditis) and thyroid cancer, necessitating extended resection and neck lymph node dissection. Indeed, it has been reported that high-volume surgeons are able to reduce the increased disease-related risk of post-thyroidectomy complications [9].

Moreover, it has also been demonstrated that high-volume surgeons and high-volume hospitals are associated with a significantly shorter hospital stay and costs [12]. One paper showed how more extended and radical thyroidectomies (i.e., total thyroidectomy plus central neck dissection) are associated with an increased rate of complications, namely prolonged hypocalcemia, in high-volume but not in low-volume centers in South Korea. However, overall, complication rate in high-volume centers was significantly lower with respect to low-volume centers, even in the case of the association of central neck dissection [27].

Several risk factors have been described for post-thyroidectomy hypocalcemia, related to the disease (including autoimmune thyroid disease, retrosternal goiter, reoperative surgery), and to the surgery itself (including extension of thyroidectomy and concomitant neck dissection) [13]. Obviously, the reason for post-thyroidectomy hypocalcemia is mainly related to parathyroid disfunction consequent to parathyroid manipulation [61]. Consequently, parathyroid identification, manipulation, and in situ preservation play a crucial role in maintaining adequate post-thyroidectomy function and are mainly dependent on surgeon experience [62]. Of note, differently from recurrent laryngeal nerve palsy, immediate, usually transient, post-thyroidectomy hypocalcemia and hypoparathyroidism do not reflect permanent hypoparathyroidism rate and are not useful tools to assess the quality of thyroidectomy and to monitor surgeons’ performance [17].

In most of the published papers focusing on correlation between volume and quality outcome of thyroid surgery, immediate complication rate is usually reported and there is no standard definition of post-thyroidectomy hypocalcemia [63]. However, from the amount of the published literature, it seems evident the main determinant of post-thyroidectomy hypoparathyroidism is surgeons’ performance, tightly related to surgeons’ experience and volume.

Some studies found postoperative hemorrhage to be inversely related to surgeon volume; however, proper risk stratification with regard to extent of resection and anticoagulative medication was not adequately addressed. Thus, postoperative hemorrhage appears to be only marginally influenced by surgeon volume. On the contrary, outcome in the event of postoperative hemorrhage would be expected to be influenced by hospital volume as more frequent practice with the imminent bleeding rate ensures adherence to emergency workflow but there are no studies addressing this issue.

Most of the authors agree that wound seroma is not influenced by the number of thyroid procedures performed. Patients operated by low-volume surgeons were more prone to have wound infection in all the studies although statistic significance was reached in only two of them. This may be due to the low prevalence of wound infection overall and the retrospective nature of the studies.

It was shown from a variety of standpoints that high-volume surgeons have better oncologic outcomes ranging from improved completeness of resection to decreased need for reoperations and even better survival (Table 9).

## Final statements

With the aim of improving the results of thyroid surgery, ESES formulates the following statements based on the current literature and accepting that the evidence level is generally low and the recommendation grades are accordingly weak:

1. There is evidence for a relationship between surgeon volume and outcomes in thyroid surgery.
2. Surgeon volume and outcome relationship exists in thyroid surgery with respect to prevalence of complications.
3. In addition to volume, cumulative experience is expected to further improve the outcome.
4. A case load of less than 25 thyroidectomies for a single surgeon per year appears reasonable for identifying a low-volume surgeon.
5. A case load of more than 50 thyroidectomies for a single surgeon per year appears reasonable for identifying a high-volume surgeon.
6. Centers with a case load higher than 100 thyroidectomies per year can be considered high-volume centers.
7. Surgeries for thyroid cancer and autoimmune thyroid disorders are predictors of increased risk of surgical morbidity and hence should be undertaken by high-volume surgeons.
8. Oncological results of thyroid cancer surgery are significantly better if performed by high-volume surgeons.

These recommendations intent to give guidance for training, certification, accreditation, and patient awareness in the realm of improved quality.
