# Correction to: Volume, outcomes, and quality standards in thyroid surgery: an evidence-based analysis—European Society of Endocrine Surgeons (ESES) positional statement

**DOI:** 10.1007/s00423-021-02257-y

**Published:** 2021-08-07

**Authors:** Kerstin Lorenz, Marco Raffaeli, Marcin Barczyński, Leyre Lorente-Poch, Joan Sancho

**Affiliations:** 1 Department of Visceral, Vascular, and Endocrine Surgery, Luther University of Halle-Wittenberg, Ernst-Grube Strasse, 40, 06120 MartinHalle an der Saale, Germany; 2 U.O.C. Chirurgia Endocrina E Matabolica, Fondazione Policlinico Universitario Agostino Gemelli IRCCS, Rome, Italy; 3 Dipartimento Universitario Di Medicina E Chirurgia Traslazionale, Università Cattolica del Sacro Cuore, Rome, Italy; 4 Department of Endocrine Surgery, Third Chair of General Surgery, Jagiellonian University Medical College, Kraków, Poland; 5 Secció del Servei de Cirurgia General de L’Hospital del Mar, Barcelona, Spain

**Correction to Langenbeck’s Archives of Surgery (2020) 405:401–425**

**https://doi.org/10.1007/s00423-020-01907-x**

The article “Volume, outcomes, and quality standards in thyroid surgery: an evidence-based analysis—European Society of Endocrine Surgeons (ESES) positional statement,” written by Kerstin Lorenz, Marco Raffaeli, Marcin Barczyński, Leyre Lorente-Poch, and Joan Sancho, was originally published Online First without Open Access. After publication in volume 405, issue 4, pages 401–425, the author decided to opt for Open Choice and to make the article an Open Access publication. Therefore, the copyright of the article has been changed to © The Author(s) 2021 and the article is forthwith distributed under the terms of the Creative Commons Attribution 4.0 International License, which permits use, sharing, adaptation, distribution, and reproduction in any medium or format, as long as you give appropriate credit to the original author(s) and the source, provide a link to the Creative Commons license, and indicate if changes were made. The images or other third party material in this article is included in the article’s Creative Commons license, unless indicated otherwise in a credit line to the material. If material is not included in the article’s Creative Commons license and your intended use is not permitted by statutory regulation or exceeds the permitted use, you will need to obtain permission directly from the copyright holder. To view a copy of this license, visit http://creativecommons.org/licenses/by/4.0/.

The original article has been corrected.

